# Effects of Copaiba oil in the healing process of urinary bladder in rats

**DOI:** 10.1590/S1677-5538.IBJU.2017.0143

**Published:** 2018

**Authors:** Denilson José Silva Feitosa, Luan Teles Ferreira de Carvalho, Ingrid Rodrigues de Oliveira Rocha, Camila Noura de Brito, Rodrigo Alencar Moreira, Charles Alberto Villacorta de Barros

**Affiliations:** 1 Laboratório de Cirurgia Experimental, Universidade Estadual do Pará, Belém, PA, Brasil; 2 Departamento de Pesquisa Cirúrgica e Experimental, Universidade Estadual do Pará, Belém, PA, Brasil; 3 Departamento de Cirurgia Experimental e Anestesiologia, Universidade Estadual do Pará, Belém, PA, Brasil

**Keywords:** Urinary Bladder, Wound Healing, Plants, Medicinal, Collagen

## Abstract

**Introduction:**

The appropriate closure of the urinary bladder is important to many urologic procedures to avoid the formation of fistulas and strictures by excessive fibrosis. This paper presents the alterations in the bladder healing process of rats after the topical use of Copaiba oil (Copaifera reticulata).

**Material and Methods:**

Ten male Wistar rats were used and randomly divided into two groups: Control group (CG): injected 1ml/kg of saline solution on the suture line; and Copaiba group (CpG): 0.63ml/kg of copaiba oil applied to the suture line. Euthanasia was performed on the seventh day after surgery. The criteria observed were adherences formation, histopathological modifications and stereology for collagen.

**Results:**

Both groups showed adhesions to the bladder, with no statistically significant difference (p=0.1481). The microscopic evaluation revealed a trend to more severe acute inflammation process on the CpG, but there was statistical difference only in the giant cells reaction (p=0.0472) and vascular proliferation (p=0.0472). The stereology showed no difference.

**Conclusion:**

The copaiba oil modified the healing process, improving the quantity of giant cells and vascular proliferation, but not interfered in the collagen physiology.

## INTRODUCTION

The appropriate closure of the urinary bladder is important to many urologic procedures to avoid the formation of fistulas and strictures by excessive fibrosis (1). Although healing occurs similarly in different tissues, some organs have their peculiarities. In the case of the urinary bladder, scarring may be impaired by the presence of urine (2). Although when compared with healing of the intestines, the bladder has a faster healing rate and tensile strength gain (3).

Regenerative medicine has been trying alternatives to common suture threads to improve the rapid regeneration of the bladder, such as adhesives and barbed sutures (4). In addition, the use of medicinal plants in bladder healing has shown interesting results in experimental research (5, 6).

The copaiba oil (Copaifera sp.) is a native Amazonic herb and has many proprieties described such as anti-inflammatory, antibiotic, wound healing and anti-neoplastic (7). The majority of these were already scientifically described in experimental and clinical research, but none of that research tested this oil on the bladder healing process. To develop knowledge about the potential benefits of this plant, we evaluated the use of copaiba oil in the healing process of the urinary bladder in rats by macro- and microscopic analysis, regarding the formation of adherences, histopathological characteristics and stereology for collagen. This paper presents the alterations on the inflammatory response and collagens physiology in the bladder healing process of rats after the topical use of copaiba oil (Copaifera reticulata).

## MATERIAL AND METHODS

### Animals

Ten male Wistar rats (Rattus norvegicus), weighing between 200g and 250g, were used in this study. The animals were housed in steel cages, cleaned twice a week, and put in a controlled environment with a temperature of 22±2°C, with adequate humidity and artificial light in a photoperiod of 12/12 hours. Water and food were offered ad libitum throughout the study. The experimental procedures started only after the approval of this research by the Ethics Committee in Animal's Use from the Pará State University by the protocol 25/2015.

### Experimental design

Rats were randomly divided into two groups, with five animals each. Groups were established as follows: Control group (cystotomy was performed followed by suture and the injection of 1mg/kg of saline solution directly on the suture. N: 5) and Copaiba group (cystotomy was performed followed by suture and the injection of 1mg/kg of copaiba oil directly on the suture. N: 5).

### Copaiba oil

The Copaiba Oil was obtained commercially from Amazon Oil^©^. The oil was extracted in the city of Itaituba, Brazil and was certified by a chemical engineer. Its composition was 45% of β-caryophyllene.

### Surgery procedure

**Figure 1:**
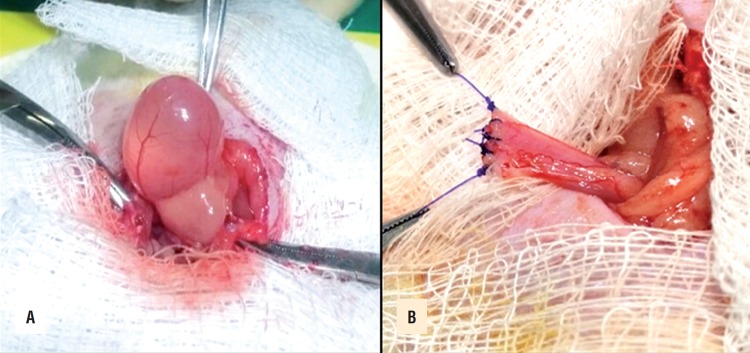
Urinary bladder before (A) and after the cystotomy and suture with PDS^©^ (B).

The animals were anesthetized by intraperitoneal injection of ketamine (70mg/kg) and xylazine (10mg/kg). Then, the rats were put in supine position on a surgical board. The trichotomy and antisepsis of the lower third of the abdominal region were performed and a median incision of 2cm made to expose the bladder (Figure-1A). Then, an 1cm cystotomy was performed and sutured with 4-0 Polydioxanone - PDS^©^ (Figure-1B). For the cystotomy instead of incising the serosa, muscular and mucosa at one time, we divided this step into two stages, reserving an incision only for the mucosa. Immediately following, either the saline solution or copaiba oil were injected, depending on the group. Then, all the animals had their abdominal wall closed with 4-0 nylon in continuous suture for the muscle-aponeurotic plane and separated for the skin.

### Macroscopic analysis

On the seventh day after surgery, the rats were euthanized by an overdose of halothane by inhalation. Then, the macroscopic analysis began with inspection of the abdominal scar and verification of presence or absence of infection in the cavity. With the abdominal wall opened, we proceeded to evaluate adhesion formation, using the Nair score (8) (Table-1).

**Table 1:** Nair score (Nair; Bath and Aurora, 1974).

Score	Morphological finds
0	Absence of adhesions
1	Single band of adhesions, between viscera, or from one viscus to abdominal wall
2	Two bands, either between viscera of rom viscera to abdominal wall
3	More than two bands, between viscera, or viscera to abdominal wall, or whole of intestines forming a mass without being adherent to abdominal wall
4	Viscera directly adherent to abdominal wall, irrespective of number and extent of adhesive bands

### Microscopic analysis

After total cystectomy, the bladder was fixed in 10% formalin by 48h and the suture threads were removed. The piece was cleaved into three parts, contemplating the border, center and incision of the bladder. The tissues were dehydrated, diaphanized in xylol and parafinized. Then, the tissues were cut into 5μm sections at 250μm intervals, mounted on slides and stained with hematoxylin/eosin and Masson's trichrome stains.

The criteria observed were acute inflammation, necrosis, foreign-body giant cell reaction, re-epithelialization and vascular proliferation in hematoxylin/eosin stain. Fibroblast and collagen growth was estimated in the Masson's trichrome stain. Each item was graded from 1 to 4, as follows: 1- absent; 2- mild; 3- moderate; 4- intense.

### Stereology

Five sections stained with Masson's trichrome were randomly analyzed from each bladder. In each section, five fields were analyzed, totaling 15 fields per bladder. The analyzed fields were digitized to a final magnification of x100 using a video camera coupled to a light microscope.

The volumetric density of the collagen fibers and muscle fibers in the muscular layer was analyzed by overlaying the M-42 grid system on the computed morphological image of the slides. The volumetric density was the relative density taken up by fibers in the tissue under examination. The stereological method determined quantitatively the parameters of the anatomical structural based on the two-dimensional thin sections, in three dimensions.

The equation $Vv=\frac{Ps}{Pp}\times 100\%$ was used to calculate the volume density of the collagen fibers, where: Vv=volumetric density, Ps=the number of structure points studied (collagen) and Pp=the number of possible test points (42 in this case).

### Statistical analysis

The statistical analysis was made in the software Bioestat^©^ 5.3. The Mann-Whitney test was used, with p <0.05 as the criterion for significance for all statistical comparisons.

## RESULTS

### Macroscopic analysis

There was no infection on the abdominal surgical wound in either group. In both the control and copaiba groups, we observed adhesion to the bladder with the omentum and the abdominal wall, with no statistically significant difference (p=0.1481). The categories of adhesions following the Nair Score found in each group are described in Table-2.

**Table 2:** Categories of adhesion according the Nair score in both groups.

	Control Group	Copaiba Group
Rat 1	1	2
Rat 2	2	1
Rat 3	1	1
Rat 4	1	2
Rat 5	1	2

Mann-Whitney test, p=0.1481

### Microscopic analysis

**Figure 2:**
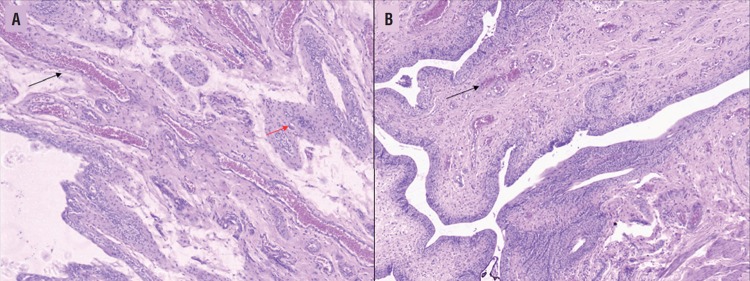
Histopathological slide of a rat urinary bladder from both the control (A) and copaiba (B) group. Note the vascular congestion and proliferation (black arrows) and the giant cell reaction (red arrow). Hematoxylin/Eosin stain 20x.

From the histopathological parameters analyzed, the only differences identified between the groups were the giant cells reaction (p=0.0472) and vascular proliferation (p=0.472) (Figure-2). The statistical analysis of histological parameters, as well as the p value for each variable, is summarized in Table-3.

**Table 3:** Statistical analysis of histological parameters by the Mann-Whitney test with the rank-sums of which group and the p value.

Histological variables	Control Group rank-sum	Copaiba group rank-sum	p
Acute inflammation	20.5	34.5	0.143
Necrosis	22.5	32.5	0.296
Giant cells reaction	18	37	0.047*
Fibroblasts	25	30	0.601
Collagen	30	25	0.601
Re-epithelialization	23	32	0.347
Vascular proliferation	18	37	0.047*

* significant statistical difference

### Stereology

**Figure 3:**
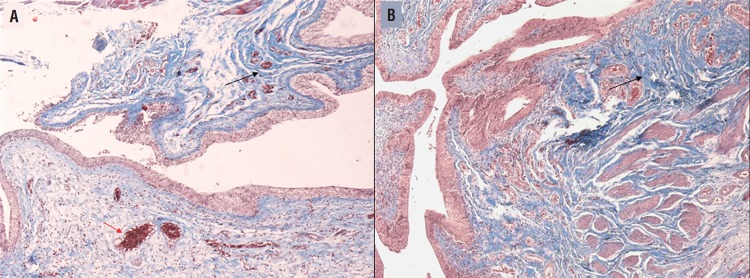
Histopathological slide of a rat urinary bladder from both the control (A) and copaiba (B) group. Note the collagen fibers (black arrow) and the vascular proliferation (red arrows). Masson's trichrome stain 20x.

In the histological sections, we observed no differences between the groups (p=0.615) (Figure-3). The fibers volumetric density in both groups is listed on Table-4.

**Table 4:** Volumetric density in percentage (Vv%) of collagen in the bladder of control and copaiba groups.

	Control Group	Copaiba Group
Mean	39	41
Standard deviation	6.38	6.34
Standard error	2.85	2.83
Minimum	29	31
Maximum	43	49

Mann-Whitney test, p=0.615

## DISCUSSION

The search for ways to improve the healing process and reduce its side effects dates to the Hippocratic era; at present, there is no therapeutic method to fully control the harmful effects of the wound healing process. The search for new drugs should be encouraged for better control of wound inflammation, which in many cases causes surgical complications.

We know that the use of copaiba in the peritoneal cavity forms adherences because copaiba oil has a corrosive effect (9). However, there was no difference between the groups regarding the formation of adherences. This data disagree with other study that used copaiba oil directly on the peritoneal cavity, which caused the copaiba group to develop greater adherences (10). This probably occurred because the dosage of copaiba oil in our study was lower and concentrated in the bladder.

Regarding the microscopic analysis, the copaiba oil modified the healing process when compared with saline solution. The copaiba group showed statistically different results in the quantity of giant cells and vascular proliferation. The giant cells are activated macrophages which play an important role in modulating the inflammation process, performing down-regulation and protecting the tissue (11).

On the other hand, the higher vascular proliferation reinforces the idea that copaiba increases local inflammation, since the factors for vascular proliferation are mainly released by activated macrophages (12). Those data suggest that the topical administration of copaiba oil may have enhanced the inflammation due to its corrosive nature, which increased the inflammatory phase of healing and delayed the beginning of the proliferative phase.

The results might be different if the copaiba oil was administrated by gavage. Yasojima et al. (13) showed that the oral administration of copaiba modulates the inflammatory response and improves wound healing in abdominal wall defects.

In relation to the amount of collagen fibers, other studies showed that copaiba oil may improve the organization of those fibers in bone regeneration when topically applied, which was not observed in our results (14). Besides having a trending to have more collagen than the control group, there was not statistical difference in the total amount, as well as occurred in the quantity of fibroblasts. Then, in this study the copaiba oil did not enhance healing.

## CONCLUSIONS

We conclude that the copaiba oil modified the healing process improving the quantity of giant cells and vascular proliferation, but not interfered in the collagen physiology.
